# Effect of pulmonary function on right heart function in Duchenne muscular dystrophy patients

**DOI:** 10.1186/1532-429X-17-S1-P279

**Published:** 2015-02-03

**Authors:** Sharath Subramanian, Kan N Hor, Wojciech Mazur, Suzanne Smart, Tam Tran, Nancy Halnon, Michael D Taylor, Linda Cripe, Subha V Raman

**Affiliations:** Cardiology, Ohio State University, Columbus, OH USA; Nationwide Children’s Hospital, Columbus, OH USA; The Christ Hospital Heart and Vascular Center, Cincinnati, OH USA; University of California Los Angeles, Los Angeles, CA USA; Cincinnati Children’s Hospital Medical Center, Cincinnati, OH USA

## Background

Duchenne muscular dystrophy (DMD) is an x-linked neuromuscular disorder in which cardiopulmonary disease leads to death in the third to fourth decade of life. Diaphragm and myocardium suffer progressive damage. While abnormalities in pulmonary and left ventricular function have been independently described, the relationship between pulmonary function and the right ventricle has not been evaluated.

## Methods

Thirteen males with DMD enrolled in an ongoing clinical trial underwent CMR with multislice short axis cine imaging, using either breathhold segmented steady-state free precession or real-time acquisition techniques. Offline analysis of right ventricular volumes and ejection fractions was performed by a single reviewer blinded to PFT results using endocardial contour delineation and Simpson's rule. Forced vital capacity (FVC%) was recorded from PFTs acquired within 10 weeks of CMR examination, and serum brain natriuretic peptide levels were measured. Subjects were classified into two groups based on having at least 80% predicted FVC.

## Results

Of the 13 subjects aged 17.5± 6.5 years (mean ± SD) who underwent CMR and concurrent FVC assessment, 11 were non-ambulatory and all subjects were on chronic oral corticosteroids at the time of imaging. Left ventricular ejection fraction (LVEF) was normal in the study group (56.9±5.3%). The right ventricular indexed stroke volume was reduced in subjects who had less than 80% predicted FVC compared to patients who had more than 80% predicted FVC (25.2±4.9 vs. 32.4±5.3 mL/m^2^; p=0.026, Figure). There was no significant difference in the LVEF between two groups (55.5±5.2 vs. 58.1± 5.6, mean ± SD; p=0.4).There was no correlation between indices of right ventricular size and systolic function and BNP levels.

**Figure 1:**
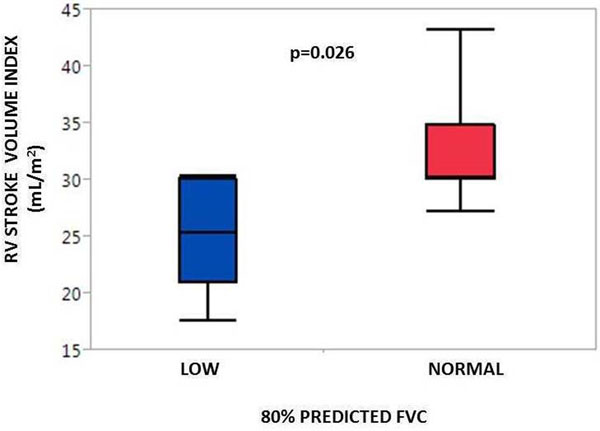
Right ventricle stroke volume vs. pulmonary forced vital capacity.

## Conclusions

Variable RV systolic function and pulmonary function were demonstrated in boys with DMD and normal LV systolic function as assessed by CMR. Abnormal pulmonary function is correlated with reduced RV stroke volume in boys with DMD and preserved LV systolic function. Further studies are needed to ascertain the prognostic significance and the influence of ventilatory support on these findings.

## Funding

This work was supported by BallouSkies.

